# NFE2L2 variations reduce antioxidant response in patients with Parkinson disease

**DOI:** 10.18632/oncotarget.7353

**Published:** 2016-02-12

**Authors:** YaXing Gui, LiShan Zhang, Wen Lv, WenMing Zhang, JinJia Zhao, XingYue Hu

**Affiliations:** ^1^ Department of Neurology, Sir Run Run Shaw Hospital, Affiliated with School of Medicine, Zhejiang University, Hangzhou, Zhejiang, China

**Keywords:** Parkinson disease, polymorphism, oxidative stress, Gerotarget

## Abstract

Oxidative stress has been recognized as a risk factor of Parkinson's disease (PD) development. We hypothesized that decreased function of the nuclear factor (erythroid-derived 2)-like 2 (NFE2L2)-antioxidant response element (ARE) pathway might predispose to Parkinsonism. A case-control study was performed between NFE2L2 Single Nucleotide Polymorphism (SNP) and PD in a cohort of 765 unrelated patients with diagnosis of PD and 489 matched normal individuals. We found that c.351T>A, D117E (*P* = 0.003, OR = 2.8) and c.351T>A, D117E (*P* = 0.012, OR = 1.9) were significantly associated with PD. The risk allele of both polymorphisms showed a high frequency in our PD sample (c.351A: 19.7% and c.423T: 7.8%). The association between both c.351T>A and c.423G>T and PD was further confirmed in an independent case-control cohort consisting of 210 individuals with PD and 148 normal controls. We further found that over expression of D117E and Q141H variants of NFE2L2 reduced target genes expression of Glutathione S-transferase Pi 1 (GSTP1), Glutathione S-transferase Mu 1 (GSTM1), and Heme oxygenase 1 (HO-1) genes. NFE2L2 D117E and Q141H impaired activation of ARE-driven transcriptional activity. Our findings indicate that NFE2L2 may play an important role in the pathogenesis of PD in Chinese populations.

## INTRODUCTION

Oxidative stress has been known as one of the pathogenic pathways contributing to the development of Parkinson's disease (PD) [1-4]. Several PD related genes such as Parkin, DJ1, and PINK1 have been discovered from GWAS studies [1, 5, 6] and demonstrated to be involved in oxidative stress processes during PD pathogenesis processes [1, 5, 6]. In addition, and Parkin, DJ1, and PINK1 have already been investigated in cellular protection against oxidative damage *in vitro* [1, 5, 6]. Further, some other studies have shown there were increased expression of oxidative markers and decreased antioxidant enzyme activities in the substantia nigra compacta (SNc) in PD subjects [1, 4-9].

Nuclear factor erythroid derived 2-like 2 (NFE2L2, also known as Nrf2) and its negative regulator protein Kelch-like ECH-associated protein 1 (Keap1) are the central redox-sensitive transcription factors controlling the oxidative stress response [1, 6, 10]. NFE2L2 binds to antioxidant response element (ARE), which leads to the expression of NADPH dehydrogenase 1 (NQO1) and heme oxygenase 1 (HO1) and then increases antioxidant capacity [11-13]. Cytoplasmic NFE2L2 level was down regulated through proteasomal degradation by Keap1 ubiquitination. NFE2L2 and NQO1 expression were reduced in striatum in MPTP-treated mice; and NFE2L2 mediated ARE activation was sufficient to produce detoxification enzymes in brain nigrostriatal dopaminergic pathway in rodent PD model [14, 15]. In addition, NFE2L2 was observed to be translocated from the cytoplasm into the nucleus in response to oxidative stress and transactivate expression of downstream genes with antioxidant activity. Furthermore, oxidative damage was abundant in Alzheimer's and Parkinson's disease [1, 16]. It is therefore to consider NFE2L2 as potential candidate gene for genetic study in PD cohorts.

Since NFE2L2 exerts a protective role for dopaminergic neuron from oxidative stress, this study was conducted to evaluate if there was an association of NFE2L2 variants with PD susceptibility in Chinese populations. Here, we performed extensive screening of NFE2L2 gene by direct sequencing to detect polymorphisms, and statistical analysis to examine its genetic effect on PD pathogenesis. We further examined the functional effect of NFE2L2 variants in regulating dysregulated oxidative stress.

## RESULTS

### NFE2L2 promoter, exonic, and intronic SNP screening

By direct DNA sequencing in twenty-five individuals (15 PD patients and 10 normal controls), we identified 10 SNPs within the NFE2L2 gene, including 3 exonic SNPs, 2 intronic SNPs, 3 promoter SNPs, and 2 SNPs in 3′ region of NFE2L2 gene. Among 10 SNPs identified, 7 SNPs were already registered in the dbSNP database, and 3 SNPs are novel (c.351T > A, c.391G > A, and c.423G > T, Table 1).

**Table 1:** Analyses of association of NFE2L2 gene polymorphisms with Parkinsonism

SNP^1^	Location	n^2^	Genotype frequency					Allele frequency		
			Genotype			*P*-value^3^	OR(95%CI)^4^	Allele	*P*-value^5^	OR(95%CI) ^4^
rs2588882,T>G MAF^6^=0.118	3′region		TT	T/G	GG		1.107 (0.768-1.451)	G		1.002 (0.699-1.345)
	Cases	765	0.737	0.204	0.059	0.574		0.161	0.978	
	Controls	489	0.722	0.205	0.073			0.175		
rs35652124,A>G MAF^6^=0.352	Promoter-653		AA	A/G	GG		1.054 (0.725-1.412)	G		1.027 (0.677-1.543)
	Cases	765	0.477	0.350	0.173	0.926		0.348	0.944	
	Controls	489	0.480	0.340	0.180			0.350		
rs6706649,G>A MAF^6^=0.078	Promoter-651		GG	G/A	AA		1.151 (0.659-1.668)	A		1.123 (0.757-1.465)
	Cases	765	0.761	0.180	0.019	0.241		0.109	0.971	
	Controls	489	0.829	0.156	0.015			0.094		
rs6721961,C>A MAF^6^=0.150	Promoter-617		CC	C/A	AA		0.945 (0.855-1.137)	A		1.011 (0.992-1.014)
	Cases	765	0.690	0.192	0.117	0.894		0.213	1	
	Controls	489	0.684	0.203	0.112			0.214		
rs2706110,C>T MAF^6^=0.31	3′region		CC	C/T	TT		1.200 (0.775-1.658)	T		1.080 (0.757-1.339)
	Cases	763	0.611	0.306	0.082	0.079		0.235	0.981	
	Controls	489	0.654	0.248	0.097			0.221		
rs10183914,C>TMAF^6^=0.256	Intron		CC	C/T	TT		1.128 (0.737-1.526)	T		1.102 (0.790-1.336)
	Cases	764	0.564	0.298	0.137	0.600		0.286	0.981	
	Controls	489	0.593	0.278	0.128			0.267		
rs1806649,C>T MAF^6^=0.138	Intron		CC	C/T	TT		1.026 (0.696-1.467)	T		1.070 (0.758-1.440)
	Cases	762	0.815	0.153	0.031	0.757		0.108	0.987	
	Controls	489	0.817	0.158	0.024			0.101		
c.351T>A,D117E	Exon3		TT	T/A	AA		2.852 (1.997-4.874)	A		1.196 (0.893-1.349)
	Cases	765	0.694	0.218	0.088	0.00267		0.197	0.00458	
	Controls	489	0.878	0.094	0.018			0.065		
c.391G>A,D131N	Exon3		GG	G/A	AA		1.102 (0.781-1.383)	A		1.176 (0.786-1.460)
	Cases	765	0.732	0.181	0.086	0.212		0.176	0.966	
	Controls	489	0.751	0.188	0.060			0.154		
c.423G>T,Q141H	Exon4		GG	G/T	TT		1.906 (1.114-3.213)	T		1.815 (1.353-3.556)
	Cases	764	0.872	0.101	0.028	0.0112		0.078	0.047	
	Controls	489	0.911	0.074	0.015			0.052		

1 SNP name for genotype in cases and controls.

2 Number of valid subjects who were successfully genotyped for each of SNP.

3 Analysis performed by a 2 × 2 table for each SNP using major homozygotes vs. others in cases and controls.

4 Reference group (controls) designated with an OR of 1.00.

5 Analysis performed by a 2 × 2 table for the number of each allele in cases and controls.

6 MAF: reported minor allele frequency of the known SNPs in NCBI dbSNP.

### Association of NFE2L2 polymorphisms with PD

**Figure 1:**
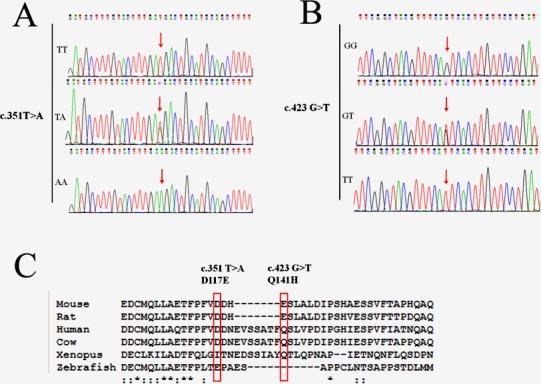
Genomic DNA sequence electropherograms of the two exonic SNPs of NFE2L2 gene are shown Panel **A.** shows c. 351T > A; Panel **B.** shows c. 423G > T. Panel **C.** shows NFE2L2 sequence alignment and exonic polymorphisms are evolutionarily conversed. Multiple sequence alignments of NFE2L2 homologous sequences in different species (Human, Cow, Mouse, Rat, Xenopus, and Zebrafish) are shown. DNA variations reported in this study are highlighted in red rectangle.

Ten identified SNPs were selected for larger scale genotyping in 765 PD patients and 489 normal controls for PD genetic association study. In our study, the genotype distribution for all SNPs was in Hardy-Weinberg equilibrium. Cases and controls were genotyped for ten SNPs including three promoter SNPs rs35652124, rs6706649, and rs6721961. We did not identify any significant association between these three promoter SNPs and PD (*P* > 0.05, Table 1). To determine whether the alleles were acting in combination, we compared compound genotypes in PD cases and normal controls. There was no difference in the frequency of four compound genotypes defined by rs35652124, rs6706649, and rs6721961. However, two exonic SNPs (c.351T > A and c.423G > T) had significant associations with PD among three exonic SNPs of the NFE2L2 gene (Figure 1A and Figure 1B and Table 1). The statistical P-values of c.351T > A and c.423G > T was 0.00267 (OR = 2.852) and 0.0112 (OR = 1.906) for genotype association study. Pairwise LD was measured to investigate the pattern of LD of the NFE2L2 locus among c.351T > A and c.423G > T. Furthermore, human 351T and 423G alleles were conserved in Xenopus, Mouse, Rat, Cow and Human (Figure 1C). These exonic substitutions just happened to be nearby the pyrimidine-rich region of the 3′splicing acceptor. Mutations within the pyrimidine-rich intronic sequence could cause human diseases. Therefore, it is of great interest and importance to examine the effect of these two polymorphisms on RNA splicing. The association between both c.351T > A and c.423G > T and PD was further confirmed in an independent case-control sample consisting of 210 cases with PD and 148 normal controls from Henan province of China (Table 2). Taking all together, these results demonstrated that exonic SNPs rather than promoter SNPs of NFE2L2 gene were associated with PD development in Chinese populations.

**Table 2:** Analysis of association of *NFE2L2* exonic SNPs with PD in an independent cohort

SNP^1^	Location	n^2^	Genotype frequency					Allele frequency		
			Genotype			*P*-value^3^	OR(95%CI)^4^	Allele	*P*-value^5^	OR(95%CI) ^4^
c.351T>A,D117E	Exon 3		TT	T/A	AA		1.921(1.098-3.363)	A		1.596(1.115-2.235)
	Cases	210	0.780	0.150	0.070	0.0048		0.145	0.042	
	Controls	148	0.890	0.080	0.030			0.070		
c.423G>T,Q141H	Exon 4		GG	G/T	TT		2.227(1.186-2.970)	T		1.027(0.834-1.253)
	Cases	210	0.815	0.109	0.076	0.043		0.065	0.397	
	Controls	148	0.909	0.069	0.022			0.057		

1 SNP name for genotype in cases and controls.

2 Number of valid subjects who were successfully genotyped for each of SNP.

3 Analysis performed by a 2 × 2 table for each SNP using major homozygotes vs. others in cases and controls.

4 Reference group (controls) designated with an OR of 1.00.

5 Analysis performed by a 2 × 2 table for the number of each allele in cases and controls.

### Reduced expression of the downstream genes of NFE2L2 with Parkinson's disease associated polymorphism

We next explored the association between exonic SNPs and NFE2L2 expression. NFE2L2 mRNA levels were determined by real-time PCR in 36 PD patients and 28 normal controls with known genotypes and available RNA samples. Compared with PD cases with c.351T > A TT genotype, individuals with heterozygous c.351T > A TA genotype had no significantly different levels of NFE2L2 (*P* = 0.17; data not shown). There was no changed expression of NFE2L2 mRNA in normal controls with c.351T > A AA genotypes compared to those of healthy subjects with c.351T > A TT genotypes (*P* = 0.38, data not shown). These results indicated that exonic polymorphisms of NFE2L2 don't change expression levels of NFE2L2.

**Figure 2:**
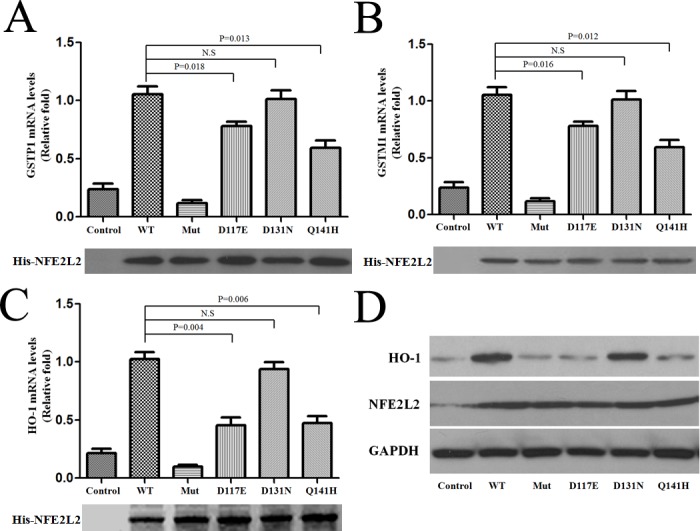
Reduced expression of the downstream genes of NFE2L2 with Parkinson's disease associated polymorphism **A.** Quantitative mRNA levels of GSTP1 in WT and mutant NFE2L2 plasmids transfected 293T cells were measured with real-time PCR. **B.** Quantitative mRNA levels of GSTM1 in WT and mutant NFE2L2 plasmids transfected 293T cells were measured with real-time PCR. **C.** Quantitative mRNA levels of HO-1 in WT and mutant NFE2L2 plasmids transfected 293T cells were measured with real-time PCR. **D.** Protein levels of HO-1 in WT and mutant NFE2L2 plasmids transfected 293T cells were measured with Western-blot. His-tagged NFE2L2 protein level was used as a loading control. All experiments were repeated three times independently.

The mRNA expression of two glutathione synthesis genes GST genes (GSTP1 and GSTM1), downstream targets of NFE2L2 activation, were reduced when over-expression of NFE2L2 variants, 117E and 141H (Figure 2A and Figure 2B). We examined another NFE2L2 target gene, Heme Oxygenase-1 (HO-1) in the 293T cells with over-expression of NFE2L2 and found that reduced expression of HO-1 mRNA and proteins when over-expression of NFE2L2 variants, 117E and 141H (Figure 2C and Figure 2D). These results indicated that novel variants, 117E and 141H, of NFE2L2 might cause impaired transcription activation function.

### Reduced activation of ARE-driven transcriptional activity by NFE2L2 variants

**Figure 3:**
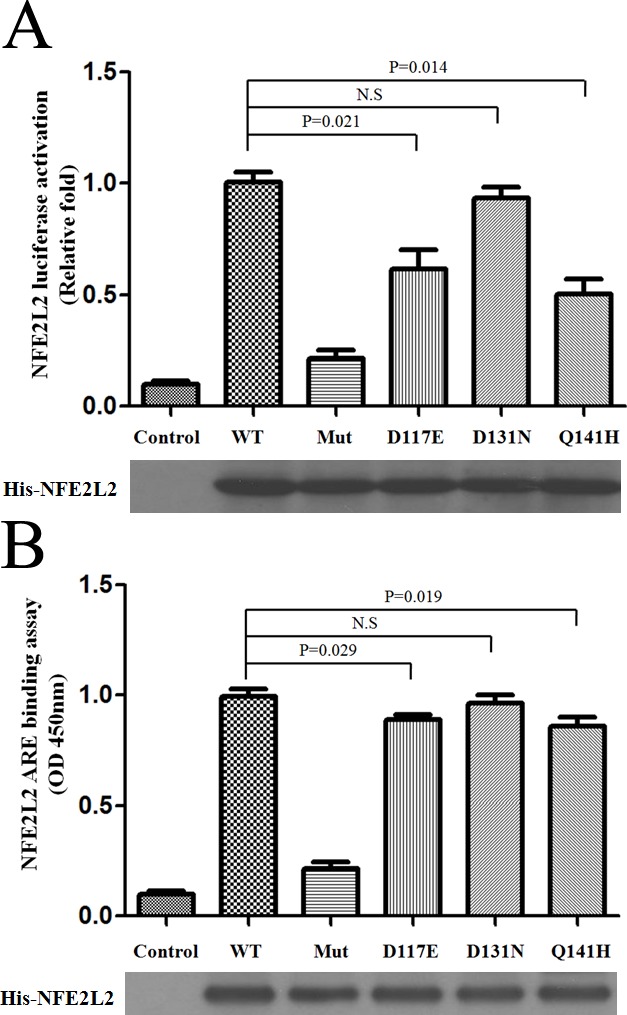
Reduced activation of ARE-driven transcriptional activity by NFE2L2 variants **A.** Cells were transfected with WT and mutant NFE2L2 plasmids as well as constructs expressing ARE-inducible Firefly luciferase. Luciferase activity was determined by measuring luminescence. **B.** NFE2L2 ARE binding was assessed in PBMC cells transfected with WT and mutant NFE2L2 plasmids. His-tagged NFE2L2 protein level was used as a loading control. Experiments in **A.** and **B.** were repeated at least three times.

To investigate mechanisms by which NFE2L2 activity may be aberrant or insufficient in neurodegenerative conditions, the effect of NFE2L2 variants on the regulation of ARE-driven signal pathway was determined. This was accompanied by an inhibition of ARE-driven luciferase activity (Figure 3A). ARE-dependent transcription was induced by NFE2L2 wide-type over-expression, while NFE2L2 variants 117E and 141H reduced induction of ARE-dependent transcription (Figure 3A). We determined the effect of NFE2L2 protein expression in whole-cell and nuclear extracts and on NFE2L2-ARE binding. NFE2L2 protein was predominantly found in the nucleus for 20 hours neither modulated its nuclear expression nor its binding to ARE consensus sequences (Figure 3B). These data demonstrated that Parkinsonism associated novel variants of NFE2L2, 117E and 141H, reduced activation of ARE-driven transcriptional activity.

## DISCUSSION

Previous studies indicated that transcription factor NFE2L2 controls antioxidant response element (ARE)-regulated antioxidant genes, contributing to an essential protective role in the brains against neurodegenerative diseases [7, 18, 19]. Therefore, NFE2L2 promises to be an attractive therapeutic target for diagnosis for PD. In this study, we provided evidence for association of novel coding SNPs of the NFE2L2 gene with PD (c.351T > A and c.423G > T) in Chinese populations with PD. We further found that over-expression of 117E and 141H variants of NFE2L2 reduced target genes expression of GSTP1, GSTM1, and HO-1 genes through impaired activation of ARE-driven transcriptional activity. Prior candidate genetic study did not discover the associations between the NFE2L2 genetic polymorphisms and PD in the Japanese and American populations, respectively [20, 21]. However, associations of NFE2L2 with PD in European populations suggested that promoter polymorphisms were protective for PD [22]. On the contrary, there was no association of these promoter SNPs and the composed haplotype with PD susceptibility in Taiwan population [22]. In our study we did not find the association between NFE2L2 promoter SNPs and PD susceptibility, either. Several factors could provide plausible explanations that we failed to observe any association with susceptibility to PD for promoter polymorphisms of NFE2L2, e.g., (1) possible genetic differences between different ethnic groups; (2) different study designs and possible biases in recruiting case and normal controls among studies; (3) and the statistical approach in the previous studies did not account for significance testing, raising the possibility of a false positive result.

Oxidative stress is considered as a common pathogenic mechanism implicated in neurodegenerative diseases including PD [7, 8, 23]. NFE2L2 played as a switch for the endogenous antioxidant response by activating its downstream target genes which regulate oxidative stress and inactivate toxic chemicals [7, 19, 23]. Reducing cellular oxidative stress occurs through an endogenous mechanism regulated at the transcriptional level of anti-oxidant genes, including glutathione-S-transferase (GST), coenzyme Q10 (Q10), NAD(P)H:quinone oxidoreductase (QR), and superoxide dismutase 1 (SOD1) which have the common promoter element called the antioxidant response element [4, 8, 11, 12, 19]. In our study, in PD patients with NFE2L2 variants (117E and 141H), we observed the effects of the NFE2L2 variants on its downstream gene expression including GSTM1 and SOD1, a major antioxidant enzyme in Parkinsonism. Therefore, genetic variants of NFE2L2 may be directly linked to reduced target gene expression in Parkinson's disease.

There are some potential reasons that how NFE2L2 variants dysregulated antioxidant response through ARE mediated downstream target genes. Base substitutions in the recognition elements of a functional ARE can influence which transcription factor dimers preferentially bind such as NFE2L2-MAF heterodimers, or MAF homodimers. Therefore, NFE2L2 variation might change its structural and functional features, and then influence its binding to ARE of its target genes, which may explain NFE2L2 as one potential candidate gene for PD susceptibility. Under basal conditions, NFE2L2 was sequestered in the cytoplasm by Keap1, which usually binds to Cul3. Therefore, NFE2L2 under basal conditions was poly-ubiquitinated and proteasomally degraded. Under conditions of oxidative stress, oxidation of Keap1 leads to dissociation of NFE2L2 which can be further activated through phosphorylation by intracellular kinases. Upon release and activation, NFE2L2 can translocate into the nucleus and combines with the bZIP family, sMaf, and binds to ARE in the promoter region of downstream target genes. Therefore, it is possible that Parkinson's disease associated NFE2L2 variation might be involved in dysregulated NFE2L2-Keap1 biological cascade and antioxidant response pathway.

There are still many limitations in this study. The method of SNP screening, especially x25 samples in discovery, might cause loss of some rare polymorphisms of NFE2L2 gene in our PD cohorts. In addition, we analyzed both promoter and open reading region SNPs and haplotype, which do not exclude any association of other region around or within the gene. Furthermore, the role of potential confounders was not evaluated in this study. Although this study did not demonstrate significant association between promoter SNPs of NFE2L2 and PD susceptibility, further search of the susceptible genes in redox pathway is warranted because of accumulating evidence of oxidative stress related pathogenesis in PD.

In conclusion, our study presents the evidence of a strong association between functional polymorphisms of NFE2L2 gene and the risk of Parkinson's disease in Chinese populations, suggesting a critical role of NFE2L2 in ROS activity in the development of Parkinson's disease. Future investigation is warranted to elucidate the detailed mechanisms of antioxidant signaling pathways in Parkinson's disease and to develop possible interventions targeting NFE2L2-ARE pathways for neurodegenerative disease therapy.

## MATERIALS AND METHODS

### Subjects

A total of 1254 study subjects were included in this study, comprising 765 patients with PD (435 males and 330 females) and 489 normal subjects without evidence of PD (354 males and 135 females). Ethnically- and sex-matched proportion between cases and normal controls was statistically matched. All PD patients were recruited from the Department of Neurology from Sir Run Run Shaw hospital affiliated with Zhejiang University School of Medicine and received a standard neurological examination as well as a psychiatric interview. The clinical diagnosis of PD was confirmed by the senior neurologist specializing in movement disorders according to UK Parkinson's disease society brain bank clinical diagnostic criteria [17]. Written informed consent for participation in the study was obtained from all subjects and the work received approval from the institution ethics committee from Sir Run Run Shaw Hospital Affiliated with School of Medicine, Zhejiang University and conformed to the tenets of the Declaration of Helsinki. The selection criteria of PD patients and normal controls in discovery panel were diagnosed the same with those of replication samples and recruited from the same areas.

Another group of 210 patients with sporadic early-onset Parkinson's disease was collected from Henan province, Central China. There were 123 male and 87 female sporadic patients with mean age of 59.3 ± 13.3 years. All patients underwent a standardized neurological examination by two movement disorder specialists. 210 PD patients were selected according to the following criteria: (1) at least two of the three cardinal motor signs (resting tremor, bradykinesia, rigidity); (2) excellent response following L-Dopa therapy; (3) absence of extensor plantar reflexes, early dementia, or early autonomic failure in the family members. A normal control group of 148 normal controls from the same geographic areas was obtained. All normal controls were free of symptoms suggestive of PD and with a negative family history of movement disorders. Informed consent was obtained before participation into our study and the work received approval from the institution ethics committee from Zhengzhou University School of Medicine in Henan province and conformed to the tenets of the Declaration of Helsinki.

### SNP screening and genotyping

The blood samples from all the participants were collected for genomic DNA extraction using the QIAamp Blood kit (Qiagen). All the PCR products of interest were Sanger-sequenced using the ABI-3100 sequencer. The results were analyzed using the ABI Prism GeneScan and Genotyper program (Applied Biosystems). Specific primers for all the exons and the flanking regions of NFE2L2 gene (NM_006164), including the promoter regions (1.5kb) were designed based on genomic sequences obtained from the GenBank DNA database.

### Amino acid sequence variation analysis

We aligned multiple sequences of NFE2L2 from different species using the Clustal W program, and predicted the secondary structure of human NFE2L2 using the PepTool Lite software (Bio tools Inc).

### Plasmids construction and site-directed mutagenesis PCR

Human NFE2L2 coding sequence was amplified and sub-cloned into pcDNA3.1 with His-tag in C-terminal. Different mutations of NFE2L2 (D117E, D131N, and Q141H) were generated according to site-mutation PCR primers using the Quick-change site-directed mutagenesis protocol (Stratagene).

### Cell culture and transfection

HEK 293T cells were cultured in DMEM medium supplemented with 10% FBS. 293T cells were provided by the Cell Bank, Shanghai Institute of Biochemistry and Cell Biology, SIBS, CAS. Transfection of recombinant WT and mutant NFE2L2 plasmids was performed in 293T cells using Lipofectamine 2000 Reagent (Life Technologies).

### Quantitative real-time PCR

Quantitative mRNA levels of NFE2L2, GSTP1, GSTM1 and HO-1 in WT and mutant NFE2L2 plasmids transfected 293T cells were measured with real-time PCR using gene-specific primers. For real-time PCR, cDNA was amplified for 40 cycles with a master mix (SYBR Green Supermix; Life technologies) using a thermo cycler (MJ). Melting curve analysis was done at the end of the reaction to assess the quality of the final PCR products. Triplicates were used for each sample, and the average C(t) value was calculated. The ΔC(t) values were calculated as C(t) sample - C(t) GAPDH. The N-fold increase or decrease in expression was calculated by the -ΔΔCt method. The subjects used for quantitative PCR examination of NFE2L2 gene were diagnosed the same as previously described.

### NFE2L2 activation and ARE reporter assay

Cells were transfected with constructs expressing ARE-inducible Firefly luciferase and constitutively active Renilla luciferase (Promega), serum-deprived, and treated as indicated in individual experiments. Luciferase activity was determined by measuring luminescence.

### NFE2L2 ARE binding assay

NFE2L2 ARE binding was assessed in PBMC lysates by using an ELISA based NFE2L2 Trans-AM transactivation kit (Active Motif). Briefly, cell lysates were loaded in a 96-well plate with immobilized oligonucleotides containing the consensus ARE to which the NFE2L2 antibody bound. This signal was detected at an OD of 450 nm after the addition of a horseradish peroxidase-conjugated secondary antibody and corresponded to the amount of NFE2L2 activation in the sample.

### Statistical analysis

Genetic association and the Hardy-Weinberg equilibrium for the distribution of genotypes were tested by χ2 analysis with Yates' correction. Odds ratios (ORs) were calculated with 95% confidence intervals (CI). The pairwise linkage disequilibrium (LD) coefficient r2 was calculated using the program Haploview. The alleles acting in combination were compared by compound genotypes in PD cases and normal controls. P value less than 0.05 was considered statistically significant.
